# Genetic Etiology Influences the Low‐Frequency Components of Globus Pallidus Internus Electrophysiology in Dystonia

**DOI:** 10.1111/ene.70098

**Published:** 2025-03-10

**Authors:** Ahmet Kaymak, Luigi M. Romito, Fabiana Colucci, Nico Golfrè Andreasi, Roberta Telese, Sara Rinaldo, Vincenzo Levi, Giovanna Zorzi, Zvi Israel, David Arkadir, Hagai Bergman, Miryam Carecchio, Holger Prokisch, Michael Zech, Barbara Garavaglia, Alberto Mazzoni, Roberto Eleopra

**Affiliations:** ^1^ The Biorobotics Institute Scuola Superiore Sant'Anna Pisa Italy; ^2^ Department of Excellence for Robotics and AI Scuola Superiore Sant'Anna Pisa Italy; ^3^ Movement Disorders Department Fondazione IRCCS Istituto Neurologico Carlo Besta Milan Italy; ^4^ Department of Neuroscience and Rehabilitation University of Ferrara Ferrara Italy; ^5^ Neurosurgery Department, Functional Neurosurgery Unit Fondazione IRCCS Istituto Neurologico Carlo Besta Milan Italy; ^6^ Department of Pediatric Neuroscience Fondazione IRCCS Istituto Neurologico Carlo Besta Milan Italy; ^7^ Department of Neurosurgery Hadassah Medical Center Jerusalem Israel; ^8^ Faculty of Medicine The Hebrew University Jerusalem Israel; ^9^ Department of Neurology Hadassah Medical Center Jerusalem Israel; ^10^ Department of Medical Neuroscience Institute of Medical Research Israel‐Canada (IMRIC), the Hebrew University‐Hadassah Medical School Jerusalem Israel; ^11^ The Edmond and Lily Safra Center for Brain Sciences The Hebrew University Jerusalem Israel; ^12^ Department of Neuroscience University of Padova Padova Italy; ^13^ Institute of Neurogenomics, Helmholtz Zentrum München Munich Germany; ^14^ Institute of Human Genetics, School of Medicine Technical University of Munich Munich Germany; ^15^ Institute for Advanced Study Technical University of Munich Garching Germany; ^16^ Unit of Medical Genetics and Neurogenetics Fondazione IRCCS Istituto Neurologico Carlo Besta Milan Italy

**Keywords:** alpha oscillations, deep brain stimulation, dystonia, electrophysiology, genetics

## Abstract

**Background:**

Elevated low‐frequency activity (4–12 Hz) within the globus pallidus internus (GPi) has been consistently associated with dystonia. However, the impacts of the genetic etiology of dystonia on low‐frequency GPi activity remain unclear; yet it holds importance for adaptive deep brain stimulation (DBS) treatment.

**Methods:**

We compared the properties of GPi electrophysiology acquired from 70 microelectrode recordings (MER) trajectories of DYT‐*GNAL*, DYT‐*KMT2B*, DYT‐*SGCE*, DYT‐*THAP1*, DYT‐*TOR1A*, DYT‐*VPS16*, and idiopathic dystonia (iDYT) patients who underwent GPi‐DBS surgery across standard frequency bands.

**Results:**

DYT‐*SGCE* patients exhibited significantly lower alpha band activity (2.97%) compared to iDYT (4.44%, *p* = 0.006) and DYT‐*THAP1* (4.51%, *p* = 0.011). Additionally, theta band power was also significantly reduced in DYT‐*SGCE* (4.42%) compared to iDYT and DYT‐*THAP1* (7.91% and 7.00%, *p* < 0.05). Instead, the genetic etiology of dystonia did not affect the spatial characteristics of GPi electrophysiology along MER trajectories.

**Conclusion:**

Considering the genetic etiology of dystonia in closed‐loop DBS treatments and utilizing theta and alpha activity for GPi stimulation may optimize clinical outcomes. MER‐based DBS lead placement can proceed independently of the underlying genetic cause.

## 1 Introduction

Dystonia is a movement disorder causing abnormal, often repetitive movements or postures due to muscle contractions, with genetic forms resulting from pathogenic mutations in causative genes leading to diverse clinical presentations [1, 2]. Deep brain stimulation (DBS) targeting the globus pallidus internus (GPi) is an effective therapy for the drug‐resistant form of dystonia [3]. Intraoperative microelectrode recordings (MERs) are routinely used to confirm the DBS lead placements, also reveal the pathophysiology of movement disorders [4, 5].

Elevated low‐frequency (4–12 Hz) activity in local field potentials (LFPs) of the GPi [6, 7] and subthalamic nucleus (STN) [8, 9] has been consistently associated with dystonia. This activity could potentially serve as a biomarker for closed‐loop DBS in dystonia [9, 10]. Therefore, examining the impact of genetic etiology on low‐frequency GPi electrophysiology could enable personalized adaptive DBS (aDBS) treatments.

Herein, we compared the properties of low‐frequency GPi activity extracted from MERs collected during GPi‐DBS surgery from patients with various genetic and idiopathic forms of dystonia. A significant reduction in low‐frequency activity (4–12 Hz) was observed in patients with DYT‐*SGCE* compared to DYT‐*THAP1* and idiopathic dystonia, highlighting the potential for personalized aDBS based on genetic factors in dystonia patients. Finally, our findings indicate that genetic etiology has no significant impact on the spatial characteristics of GPi electrophysiology. Therefore, MER‐based DBS lead placement can be performed independently of the genetic etiology of dystonia.

## 2 Materials and Methods

The study was conducted with genetic and idiopathic dystonia patients who underwent bilateral GPi‐DBS surgery under propofol and remifentanil anesthesia (Table S1) at Fondazione IRCCS Istituto Neurologico Carlo Besta. Before 2000, only *TOR1A* was tested in patients. Between 2000 and 2015, individuals who were negative for *TOR1A* were retested via Sanger sequencing as new genes were identified. Since 2015, an NGS‐customized dystonia gene panel has been employed [11], (Table S1).

The surgeries were conducted bilaterally and under stereotactic conditions with the Leksell (Elekta Inc.) or Maranello (Eidos22) frames. A thorough description of our standard surgical procedure is available elsewhere [12]. Identification of the nuclei borders, the sensorimotor GPi, and MER depths was performed by using the Distal Atlas [13] in Montreal Neurological Institute space (*p* > 0.5 threshold) with Lead DBS v2.3 [14] and an expert electrophysiologist. At the preoperative and postoperative follow‐up evaluations, the Burke–Fahn–Marsden Dystonia Rating Scale (BFMDRS) was employed to assess the motor severity. The demographic and clinical profiles of the patients are presented in Table 1.

**TABLE 1:** Demographic, genetic, and clinical data of the cohort patients. The table reports the demographic and genetic data of the enrolled patients. In addition, data on the motor component of the Burke–Fahn–Marsden Dystonia Rating Scale (BFMDRS) at preoperative and 1‐year follow‐up following the deep brain stimulation (DBS) surgery are reported. Abbreviations: F, female; M, male.

Id	Gene	Sex	Age onset	Age at surgery	Main body part	Pattern	Preoperative BFMDRS‐M						Postoperative 1Y‐FU BFMDRS‐M					
							Total	Arms	Cranial	Neck	Legs	Trunk	Total	Arms	Cranial	Neck	Legs	Trunk
P1	*GNAL*	F	37	42	Cervical	Phasic	26	8	0	6	0	12	22	8	0	6	0	8
P2	*GNAL*	F	42	63	Cervical	Phasic	24	10	6	6	0	2	14	5	3	4	0	2
P3	*KMT2B*	M	28	45	Trunk	Tonic	27	4	3	8	0	12	10	1	0	3	0	6
P4	*KMT2B*	M	8	16	Upper	Phasic	28	13	0	6	9	0	14	5	0	3	6	0
P5	*SGCE*	M	2	48	Upper	Phasic	11.5	6	1.5	1	2	1	10.5	5	1.5	1	2	1
P6	*SGCE*	M	3	17	Upper	Phasic	16	7	0	4	4	1	2.5	1	0	0.5	1	0
P7	*THAP1*	F	40	14	Cervical	Tonic	17	4	3	8	1	1	20	2	8	8	1	1
P8	*THAP1*	F	7	32	Trunk	Tonic	32.5	4	4	0.5	12	12	5	0	0	0	3	2
P9	*THAP1*	M	6	46	Cervical	Tonic	45	4	6	8	15	12	20	2	1.5	4.5	3	9
P10	*THAP1*	M	14	43	Cervical	Tonic	60.5	13	12.5	8	15	12	31	11	4.5	4.5	2	9
P11	*TOR1A*	M	14	49	Upper	Phasic	30.5	20	0.5	4	5	1	24.5	16	0.5	4	3	1
P12	*TOR1A*	M	13	9	Lower	Tonic	29.5	0	0	0.5	20	9	4	0	0	0	2	2
P13	*TOR1A*	M	10	33	Upper	Tonic	25	13	1	0	9	2	18	10	1	0	5	2
P14	*TOR1A*	F	7	47	Lower	Tonic	43	14	0	0	25	4	4	2	0	0	2	0
P15	*TOR1A*	M	12	44	Cervical	Tonic	36	16	0	6	6	8	19	7	0	6	2	4
P16	*TOR1A*	M	10	9	Lower	Tonic	23.5	7	0	0.5	13	3	10	4	1.5	0.5	2	2
P17	*TOR1A*	F	11	23	Upper	Tonic	59	24	2.5	0.5	20	12	17.5	2	1	0.5	6	8
P18	*TOR1A*	M	8	43	Trunk	Tonic	34	0	0	0	18	16	3	0	0	0	2	1
P19	*VPS16*	F	12	49	Cranial	Tonic	23	7	11	2	2	1	13.5	7	4	0.5	0	2
P20	*VPS16*	M	15	45	Cervical	Phasic	32	12	6	6	2	6	15.5	3	5	4.5	1	2
P21	*VPS16*	M	14	24	Trunk	Tonic	52.5	20	0.5	6	14	12	25.5	12	0.5	6	5	2
P22	IDIOPATHIC	F	42	38	Cervical	Phasic	31	10	5	12	2	2	17	6	2	6	2	1
P23	IDIOPATHIC	M	3	57	Cranial	Tonic	45	2	28	3	0	12	15	3	4	6	0	2
P24	IDIOPATHIC	F	61	49	Cranial	Tonic	45	2	28	3	0	12	15	3	4	6	0	2
P25	IDIOPATHIC	F	35	30	Cervical	Phasic	32.5	2	8.5	8	6	8	5	1	0	3	0	1
P26	IDIOPATHIC	M	39	60	Cervical	Tonic	50	28	2	8	0	12	9	5	0	4	0	0
P27	IDIOPATHIC	M	35	31	Cervical	Tonic	40.5	18	10.5	6	2	4	19	10	6.5	1.5	0	1
P28	IDIOPATHIC	M	19	42	Cervical	Phasic	35.5	4	8.5	8	3	12	19	0	2	6	3	8
P29	IDIOPATHIC	F	51	63	Cervical	Tonic	26	2	0	6	6	12	21	2	0	4	6	9
P30	IDIOPATHIC	M	3	45	Trunk	Tonic	43	10	14	6	4	9	33	8	11	4	4	6

We adopted the methodology used to compare STN electrophysiology in monogenetic and idiopathic forms of Parkinson's disease (PD) [15]. Briefly, we divided MERs into 50 ms segments, computed the root mean square (RMS), and labeled segments stable if their RMS values were within three standard deviations of the median RMS. The longest stable section of each recording was selected for further analysis [16]. To estimate the power spectral density (PSD), we rectified the stable raw signal and subtracted the mean to reveal the low‐frequency envelope. The PSD was estimated with a resolution of 1/3 Hz and normalized to the total power within the analysis range (2–200 Hz) to mitigate the influence of varying RMS values across patients [16]. The length of the detected GPi region was normalized to 1, where 0 represents the GPi entry [15]. Trajectories were included in analyses if they had a minimum GPi length of 2 mm and recordings from at least four distinct depths (Figure S1). Theta (4–8 Hz), alpha (8–12 Hz), beta (12–30 Hz), and gamma (30–100), band activity was extracted using a four‐pole Butterworth band‐pass filter. The fraction of power and the ratio between these bands were measured as electrophysiological features.

Kruskal–Wallis and post hoc Dunn's test with Holm–Bonferroni correction were used for group comparison. Spearman's correlation with permutation testing was used to assess the significance of linear relationships between variables, with Benjamini/Hochberg (FDR) correction applied to the *p* values for measured correlations. All tests were two‐tailed, and statistical significance was defined as *p* ≤ 0.05. We employed a linear mixed model (LMM) with the Wald test to investigate the interaction between normalized depths and spectral properties within the GPi by considering genetic factors as random effects. This approach allowed us to assess the influence of GPi depth on spectral properties while accounting for random variation from genetic factors and individual differences.

## 3 Results

**FIGURE 1:**
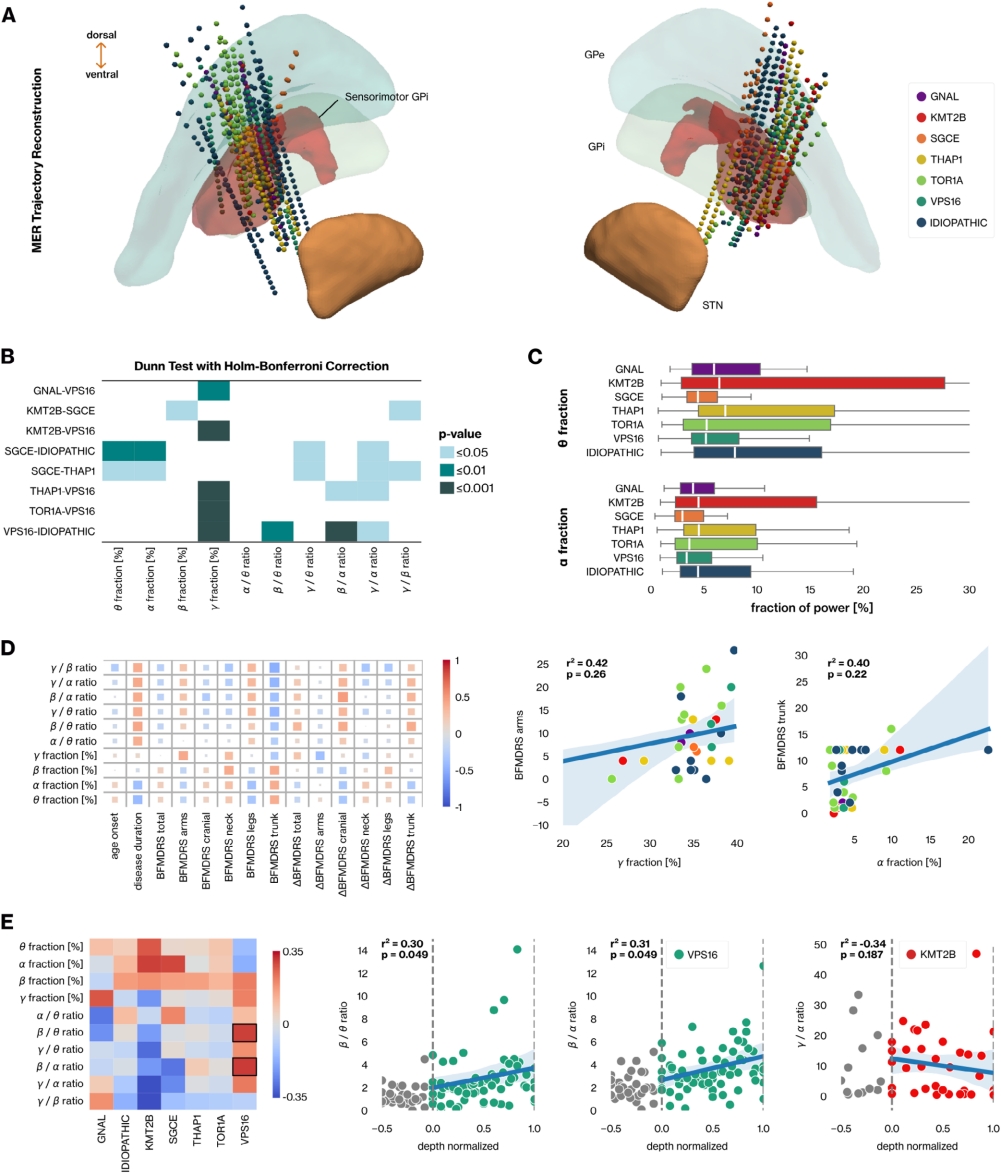
Electrophysiological characterization of power spectral properties of GPi for genetic and idiopathic dystonia syndromes. (A) Reconstruction of MER trajectories in MNI space, color‐coded according to genetic etiology. (B) Statistical comparison of power spectral features was performed with Dunn's test with Holm–Bonferroni correction; significant results are indicated by color coding. (C) Box plots displaying the distributions of theta and alpha power fraction across dystonia groups. (D) Correlation matrix depicting linear relationship between spectral and clinical features, as determined by Spearman's correlation coefficient. The magnitude of each correlation score is indicated by the size and color of the marker. Regression plots highlight significant correlations between selected feature pairs, with scatter color‐coded based on the genetic etiology. (E) Heatmap showing the correlation between normalized depth levels and electrophysiological features across patient groups. Regression plots depict the behavior of selected electrophysiological features along normalized depth levels, with the degree of correlation indicated by Spearman's correlation coefficient.

In total, 597 MERs were collected from 70 trajectories across 30 patients in the cohort (Table S2). All analyzed MER trajectories passed through the sensorimotor portion of the GPi. Therefore, we do not anticipate spatial confounders affecting the electrophysiology (Figure 1A).

Elevated relative power in the theta and alpha bands compared to the baseline (median power of the whole spectrum up to 100 Hz) was observed across dystonia syndromes (Figures S2 and S3). We detected statistically significant differences between patient groups in all spectral features except for the alpha/theta ratio (*p* ≤ 0.05, Kruskal–Wallis test with Holm–Bonferroni correction) (Table S4). The fraction of alpha band activity remained significantly lower for DYT‐*SGCE* (2.97%) compared to iDYT (4.44%, *p* = 0.006, Dunn's test with Holm–Bonferroni correction) and DYT‐*THAP1* (4.51%, *p* = 0.011) patients (Figure 1B). Similarly, the fraction of power in the theta band was significantly lower in DYT‐*SGCE* (4.42%) compared to the iDYT (7.91%, *p* = 0.002) and DYT‐*THAP1* (7.00%, *p* = 0.019) (Figure 1B,C). The fraction of gamma power for the DYT‐*VPS16* group (37.04%) was significantly higher than the remaining groups, apart from DYT‐*SGCE* (Figure 1B).

We investigated whether the observed electrophysiological differences were linked to motor symptom severity rather than genetic etiology. This was accomplished by calculating Spearman's correlation between the median values of electrophysiological features for patients and their corresponding baseline BFMDRS scores, as well as the percentage change observed in these scores between the preoperative and postoperative evaluations (Figure 1D). We observed a moderate effect size (0.4–0.6) in the relationship between certain clinical and spectral feature pairs, but none reached statistical significance (*p* > 0.05, Spearman's correlation with FDR correction) (Figure 1D). Although no significant correlation was found, the power spectral characteristics of the GPi in iDYT cases appeared to be more associated with disease severity compared to genetic dystonia syndromes (Figure S4).

Additionally, we sought to elucidate the spatial characteristics of GPi electrophysiology along MER trajectories by measuring the linear relationship between the normalized depths (GPi entry = 0, GPi exit = 1) and spectral features. The beta/theta and beta/alpha ratios (*r*_*s*_ = 0.30, *p* = 0.049) demonstrated significant linear characteristics between the borders of the GPi in DYT‐*VPS16* patients (Figure 1E). Finally, no evidence was found to support the role of genetic etiology in spatially modulating the electrophysiological properties of MER recordings within the GPi (LMM, Wald test with Holm–Bonferroni correction, *p* > 0.05) (Table S5).

## 4 Discussion

Here, we presented the potential of dystonia genetic etiology to influence the low‐frequency components of GPi electrophysiology. Reductions in theta and alpha band activities were observed in DYT‐*SGCE* compared to DYT‐*THAP1* and iDYT patients. These differences cannot be attributed to the severity of dystonia, as no significant correlation was found between these components and the severity of motor symptoms. We further demonstrated that the genetic etiology of dystonia is irrelevant to MER‐based lead localization in GPi‐DBS surgeries, as the spatial characteristics of spectral features remain consistent when genetic factors are treated as random effects in LMM.

Weill and colleagues proposed that genetic heterogeneity in PD is not associated with robust electrophysiological differences in STN [15]. In our case, we found significant differences between the low‐frequency activity of DYT‐*SGCE* from DYT‐*THAP1* and iDYT in the GPi. It has been proposed that distinct oscillatory circuit disruptions may underlie dystonia and parkinsonism, even in the same anatomical structure [9]. In this context, the effects of genetic etiology on different movement disorders and brain regions can exhibit differences. Our results align with this study, as we did not observe any impact of genetic etiology on the spatial characteristics of GPi electrophysiology.

We previously analyzed the pallidal single‐unit activity in a larger genetic dystonia cohort [17], including the patients analyzed here. We observed convergence among dystonia genes toward either strong bursts or tonic behavior, with *SGCE* and *THAP1* exhibiting opposite behaviors. This is consistent with the differing low‐frequency activity of the GPi associated with these two genes at the population level in the present work [17].

It should be noted that our study has several limitations. The limited sample size may impact the findings, though the high number of single MER epochs could mitigate this to some extent. The limited number of trajectories per patient prevented a consistent evaluation of potential spatial confounding factors along the anteroposterior and mediolateral directions. Lastly, electrophysiological activity recorded from the GPi using microelectrodes reflects considerably smaller neuronal populations compared to LFPs recorded with DBS macroelectrodes. Therefore, the potential effects of genetic etiology should also be investigated in LFP recordings.

In conclusion, we suggest the genetic etiology may potentially impact low‐frequency activity within the GPi, especially in patients with DYT‐*SGCE*, where these bands seem to be underrepresented. An adaptive DBS paradigm for dystonia based on the spectral fluctuations of recorded activity should then take into account potential differences between genetic dystonia profiles. However, further studies with larger sample sizes and a broader range of dystonia genes are needed to evaluate the clinical relevance of our hypothesis.

## Author Contributions

**Ahmet Kaymak:** conceptualization, methodology, software, data curation, investigation, validation, formal analysis, visualization, project administration, writing – original draft, writing – review and editing. **Luigi M. Romito:** conceptualization, methodology, data curation, validation, supervision, project administration, resources, writing – original draft, writing – review and editing. **Fabiana Colucci:** data curation, investigation, writing – review and editing. **Nico Golfrè Andreasi:** data curation, writing – review and editing. **Roberta Telese:** data curation. **Sara Rinaldo:** data curation. **Vincenzo Levi:** data curation, writing – review and editing. **Giovanna Zorzi:** data curation. **Zvi Israel:** supervision. **David Arkadir:** supervision, writing – review and editing. **Hagai Bergman:** supervision. **Miryam Carecchio:** supervision. **Holger Prokisch:** supervision. **Michael Zech:** supervision, writing – review and editing. **Barbara Garavaglia:** data curation, investigation, validation. **Alberto Mazzoni:** conceptualization, methodology, validation, supervision, funding acquisition, project administration, writing – original draft, writing – review and editing. **Roberto Eleopra:** supervision.

## Ethics Statement

The study was approved by the Ethical Committee of Fondazione IRCCS Istituto Neurologico Carlo Besta.

## Consent

Informed consent was obtained from patients and their legal representatives.

## Conflicts of Interest

The authors declare no conflicts of interest.

## Supporting information


Data S1.

## Data Availability

The processed spectral datasets (both spectral features and normalized PSD) from MER data supporting the findings of this study are openly available in Zenodo at https://doi.org/10.5281/zenodo.14800798 in a tabular and fully anonymized format. The complete code for data processing, descriptive analyses, and plotting is available online on the GitHub repository: https://github.com/ahmetofficial/Genetic‐DYT‐GPi‐Spectral‐Activity/.
