# Supplementary material for: Genetic Etiology Influences the Low‐Frequency Components of Globus Pallidus Internus Electrophysiology in Dystonia
Source: Eur J Neurol. 2025 Mar 10;32(3):e70098. doi: 10.1111/ene.70098 (PMC11891763; doi:10.1111/ene.70098)
Supplement: Supplementary file 1 — Data S1. [file ENE-32-e70098-s001.docx]

**Supplementary Table 1. Genetic profile and anesthetic procedure followed during DBS surgery in the studied population.** Variant classifications of mutations were made according to Franklin and ACMG criteria. The following data on the medications used during anesthesia were reported: most patients received general anesthesia comprising propofol(mg/kg/h) and/or remifentanil(µg/kg/min).

*Only one round of general anesthesia was achieved with 1 µg/kg/h of dexmedetomidine.

| ID | Gene | Variant | Franklin-ACMG  Pathogenicity Classification | Anesthetics  (remifentanil/  propofol) | |
| --- | --- | --- | --- | --- | --- |
| Pat1 | *GNAL* | c.462_463delGA p.Lys155Asnfs*9 ex3 | Pathogenic | 0.30 | 1.60 |
| Pat2 | *GNAL* | c.859G>A p.Asp287Asn ex8 | VUS | 0.60 | 2.00 |
| Pat3 | *KMT2B* | c.4561C>T p.Arg1521Trp | VUS | 0.30 | 2.00 |
| Pat4 | *KMT2B* | c.2240A>G p.Gln747Arg ex3 | VUS | 0.60 | 0.50 |
| Pat5 | *SGCE* | c.1037+3delGTGA | Likely Pathogenic | 0.40 | 1.50 |
| Pat6 | *SGCE* | c.233-1G>T | Likely Pathogenic | 0.50 | 1.20 |
| Pat7 | *THAP1* | c.70_71+8delGTCCTCACTT | Likely Pathogenic | 0.40 | 1.60 |
| Pat8 | *THAP1* | c.70_71+8delGTCCTCACTT | Likely Pathogenic | 0.60 | 1.80 |
| Pat9 | *THAP1* | c.464A>C p.Gln155Pro ex.3 | VUS | 0.70 | 1.00 |
| Pat10 | *THAP1* | c.238A>G p.Ile180Val | Likely Pathogenic | 0.50 | 2.00 |
| Pat11 | *TOR1A* | c.904_906delGAG p.del302Glu | Pathogenic | 0.50 | 1.90 |
| Pat12 | *TOR1A* | c.904_906delGAG p.del302Glu | Pathogenic | 0.50 | 1.70 |
| Pat13 | *TOR1A* | c.904_906delGAG p.del302Glu | Pathogenic | 0.00 | 1.50 |
| Pat14 | *TOR1A* | c.904_906delGAG p.del302Glu | Pathogenic | 0.40 | 1.30 |
| Pat15 | *TOR1A* | c.904_906delGAG p.del302Glu | Pathogenic | 0.00 | 0.00* |
| Pat16 | *TOR1A* | c.904_906delGAG p.del302Glu | Pathogenic | 0.70 | 1.10 |
| Pat17 | *TOR1A* | c.904_906delGAG p.del302Glu | Pathogenic | 0.80 | 1.50 |
| Pat18 | *TOR1A* | c.904_906delGAG p.del302Glu | Pathogenic | 0.50 | 2.00 |
| Pat19 | *VPS16* | c.2170_2171delAA | Likely Pathogenic | 0.80 | 1.80 |
| Pat20 | *VPS16* | c.2140C>T p.Gln714* ex21 | Likely Pathogenic | 0.30 | 1.60 |
| Pat21 | *VPS16* | c.1939C>T p.Arg647* ex20 | Pathogenic | 0.60 | 2.00 |
| Pat22 | x | x | x | 0.80 | 1.80 |
| Pat23 | x | x | x | 0.60 | 1.40 |
| Pat24 | x | x | x | 0.50 | 2.50 |
| Pat25 | x | x | x | 0.50 | 1.50 |
| Pat26 | x | x | x | 0.50 | 2.00 |
| Pat27 | x | x | x | 0.10 | 1.00 |
| Pat28 | x | x | x | 0.50 | 1.30 |
| Pat29 | x | x | x | 0.60 | 1.50 |
| Pat30 | x | x | x | 0.10 | 0.80 |

**Supplementary Table 2**. The number of patients, trajectories, and MER recordings for each dystonia group in our cohort.

|  | Patient Count | Trajectory Count | MER Count |
| --- | --- | --- | --- |
| iDYT | 9 | 21 | 205 |
| DYT-*TOR1A* | 8 | 15 | 115 |
| DYT-*THAP1* | 4 | 12 | 94 |
| DYT-*VPS16* | 3 | 7 | 72 |
| DYT-*SGCE* | 2 | 6 | 42 |
| DYT-*KMT2B* | 2 | 5 | 36 |
| DYT-*GNAL* | 2 | 4 | 33 |
|  | 30 | 70 | 597 |

**Supplementary Figure 1**. The distribution of trajectory length of microelectrode recordings among genetic dystonia syndromes and idiopathic dystonia patients.


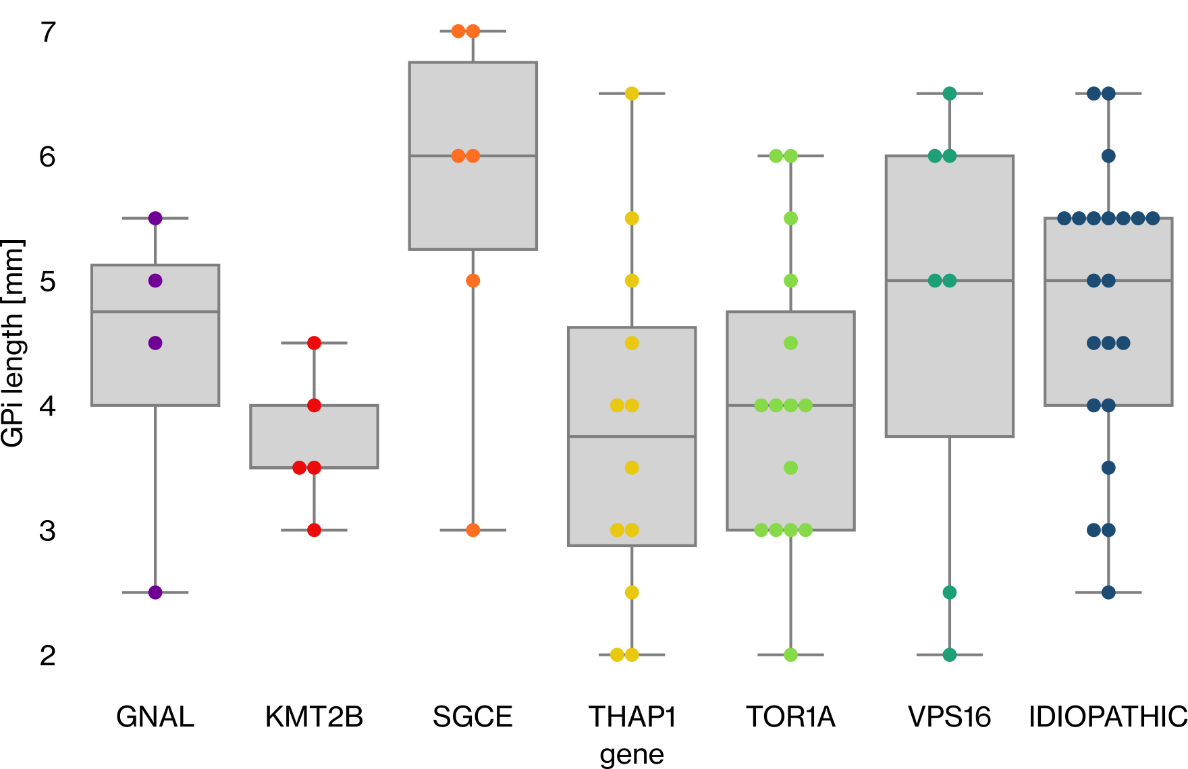


**Supplementary Table 3**. The statistical comparison of the trajectory lengths between dystonic patient groups. The comparison is made with Dunn’s test with Holm-Bonferroni correction.

|  | DYT-*GNAL* | DYT-*KMT2B* | DYT-*SGCE* | DYT-*THAP1* | DYT-*TOR1A* | DYT-*VPS16* | iDYT |
| --- | --- | --- | --- | --- | --- | --- | --- |
| DYT-*GNAL* | 1.00 | 1.00 | 1.00 | 1.00 | 1.00 | 1.00 | 1.00 |
| DYT-*KMT2B* | 1.00 | 1.00 | 0.33 | 1.00 | 1.00 | 1.00 | 1.00 |
| DYT-*SGCE* | 1.00 | 0.33 | 1.00 | 0.17 | 0.33 | 1.00 | 1.00 |
| DYT-*THAP1* | 1.00 | 1.00 | 0.17 | 1.00 | 1.00 | 1.00 | 0.70 |
| DYT-*TOR1A* | 1.00 | 1.00 | 0.33 | 1.00 | 1.00 | 1.00 | 1.00 |
| DYT-*VPS16* | 1.00 | 1.00 | 1.00 | 1.00 | 1.00 | 1.00 | 1.00 |
| iDYT | 1.00 | 1.00 | 1.00 | 0.70 | 1.00 | 1.00 | 1.00 |

**Supplementary Table 4. Results of the Kruskal-Wallis test comparing patient groups. T**he p-values presented have been corrected using the Holm-Bonferroni correction.

| features | p-values |
| --- | --- |
| *θ* fraction [%] | **0.00880** |
| *α* fraction [%] | **0.00880** |
| *β* fraction [%] | **0.01315** |
| *γ* fraction [%] | **0.00001** |
| *α/θ* ratio | 0.08440 |
| *β/θ* ratio | **0.00348** |
| *γ/θ* ratio | **0.01315** |
| *β/α* ratio | **0.00098** |
| *γ/α* ratio | **0.01229** |
| *γ/β* ratio | **0.02530** |


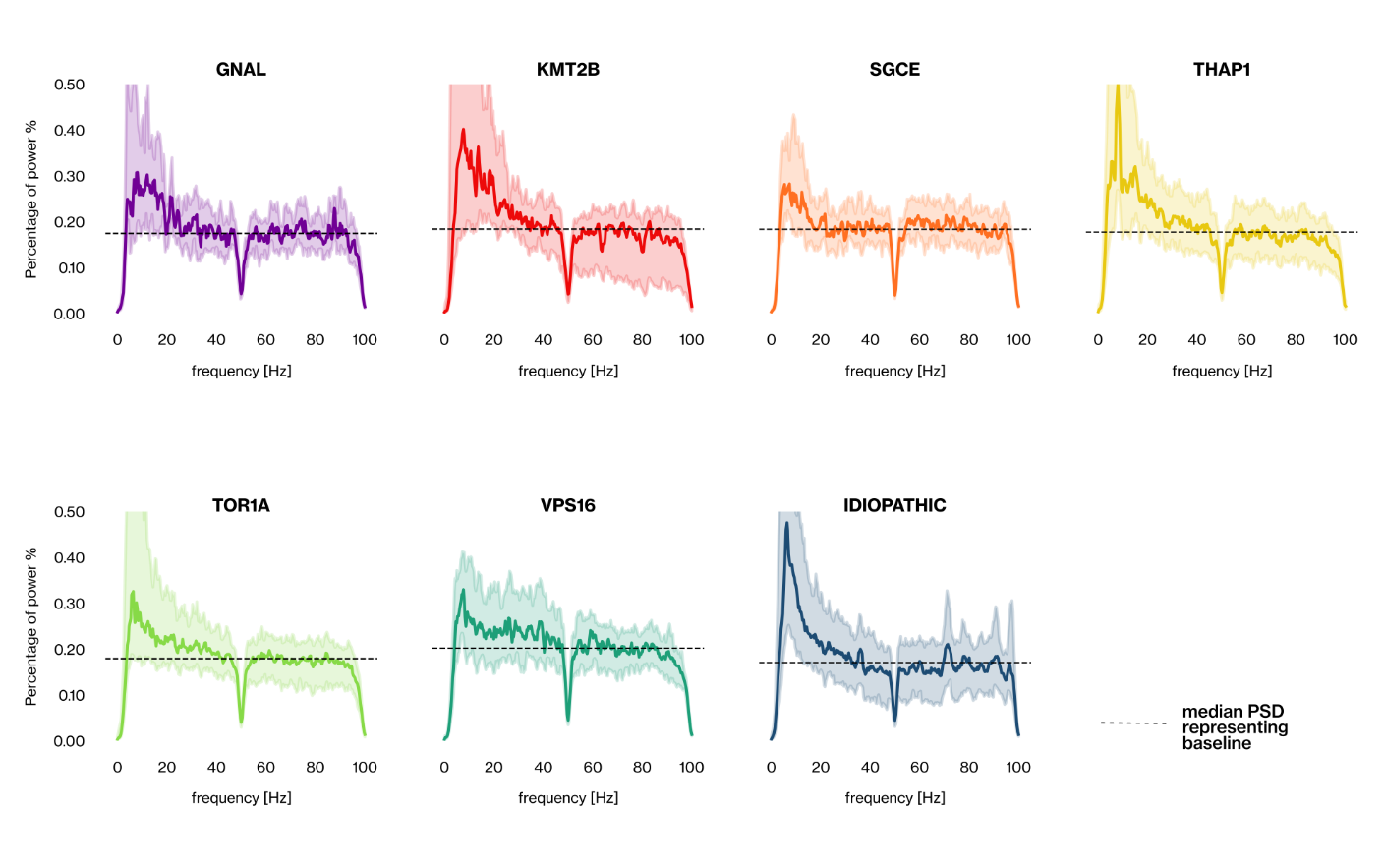
**Supplementary Figure 2**. The power spectrum of GPi activity between 0-100 Hz is compared across genetic and idiopathic dystonic groups. The line plot depicts the median proportion of power across frequencies, while the shaded area around the line represents the interquartile proportion of the power in each frequency.

**Supplementary Figure 3**. The average power spectrum density (PSD) presented heightened θ and α activity in all genetic dystonia syndromes.

**
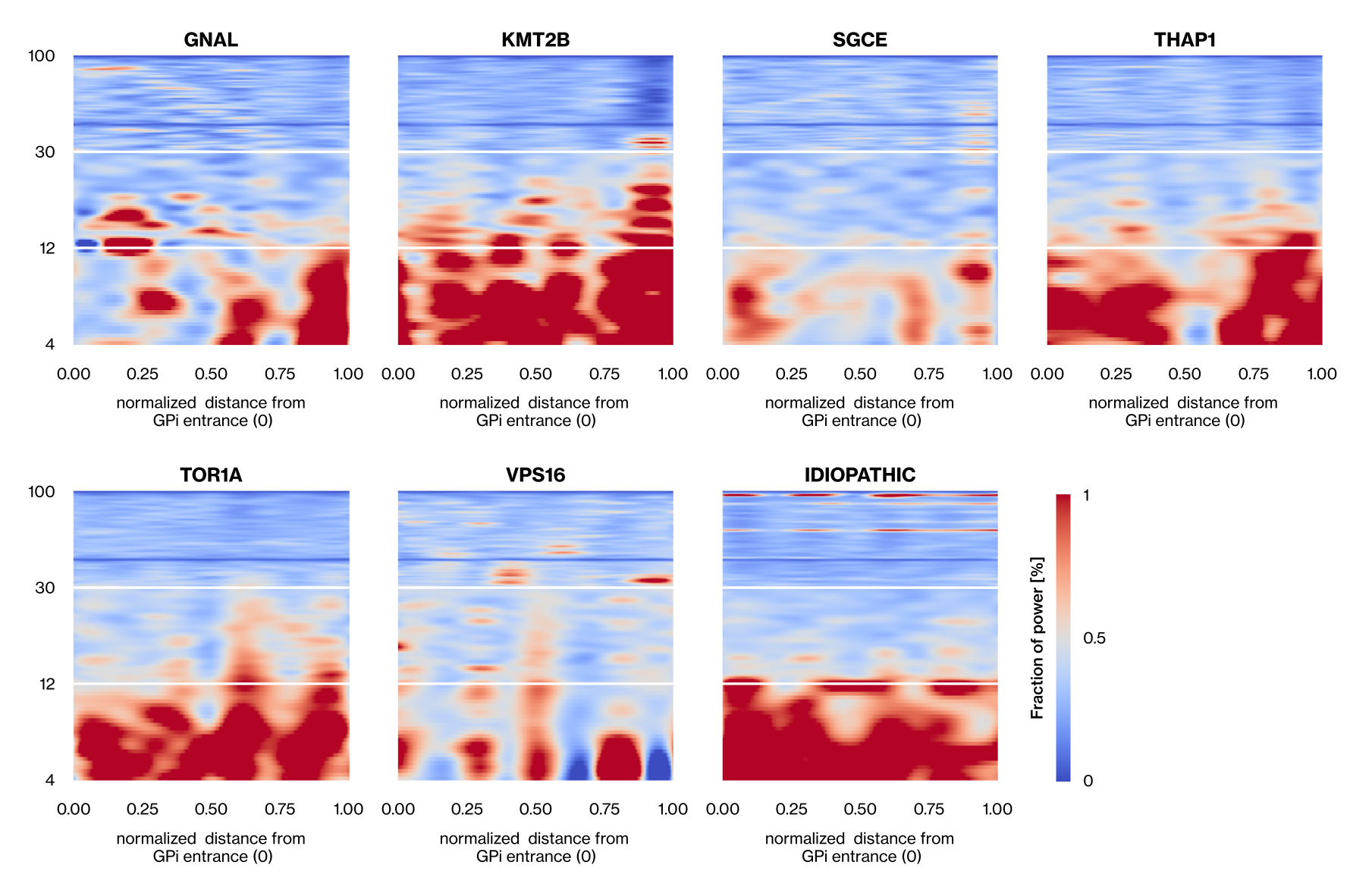
**

**Supplementary Figure 4**. The results of Spearman's Rank correlation coefficient measurements between clinical and electrophysiological features of genetic and idiopathic dystonia patients.


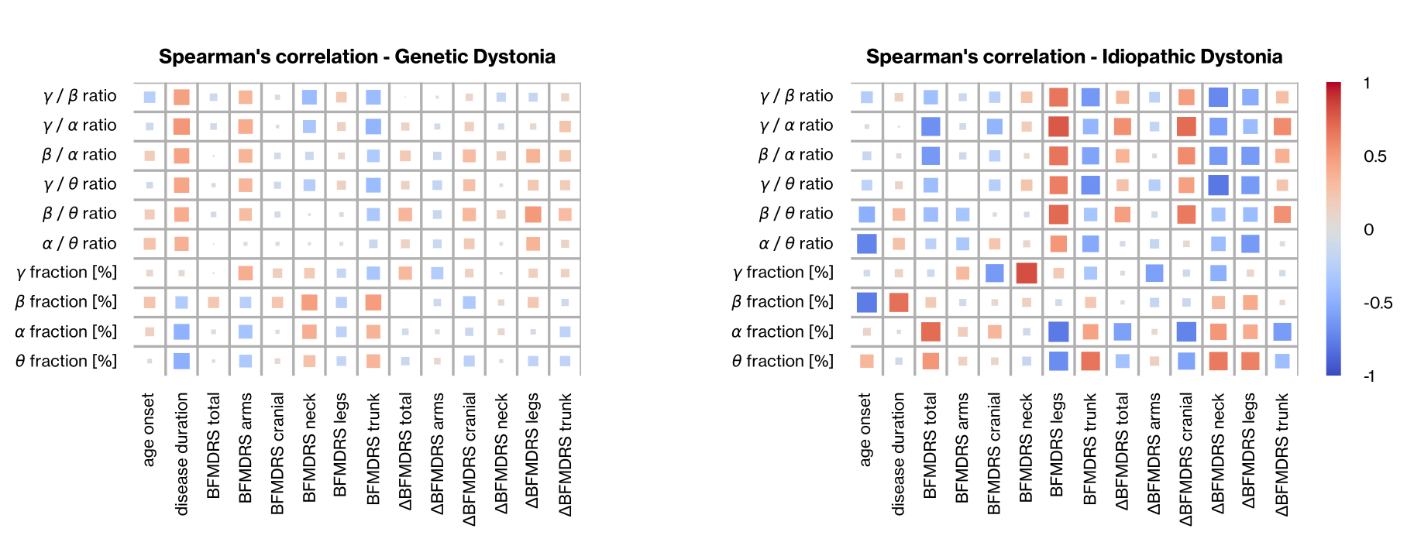


**Supplementary Table 5.** The probability values of fitted linear mixed models, with genetic factors as random effects, for each electrophysiological feature.

| features | p-values |
| --- | --- |
| *θ* fraction [%] | 1.0 |
| *α* fraction [%] | 1.0 |
| *β* fraction [%] | 0.099 |
| *γ* fraction [%] | 1.0 |
| *α/θ* ratio | 1.0 |
| *β/θ* ratio | 1.0 |
| *γ/θ* ratio | 1.0 |
| *β/α* ratio | 1.0 |
| *γ/α* ratio | 1.0 |
| *γ/β* ratio | 0.099 |

**Supplementary Table 6.** **Comparison of patients having phasic or tonic dystonic movements. The Kruskal-Wallis test with** Holm-Bonferroni correction was used to **compare two groups.**

Δ the percentual change between postoperave 1-year and baseline evaluations.

| features | p-values |
| --- | --- |
| ΔBFMDRS total | 0.842677 |
| ΔBFMDRS arms | 1.000000 |
| ΔBFMDRS cranial | 1.000000 |
| ΔBFMDRS neck | 1.000000 |
| ΔBFMDRS leg | 0.861676 |
| ΔBFMDRS trunk | 1.000000 |
